# Editorial: Recent advances in image fusion and quality improvement for cyber-physical systems

**DOI:** 10.3389/fnbot.2023.1201266

**Published:** 2023-05-04

**Authors:** Xin Jin, Jingyu Hou, Wei Zhou, Shin-Jye Lee

**Affiliations:** ^1^School of Software, Yunnan University, Kunming, China; ^2^School of Information Technology, Deakin University, Geelong, VIC, Australia; ^3^Institute of Technology Management, National Chiao Tung University, Hsinchu, Taiwan

**Keywords:** artificial neural networks, embedded learning system, feature extraction, image quality improvement, image fusion, robot vision

Multi-source visual information fusion and quality improvement can help the robotic system to perceive the real world, and image fusion is a computational technique fusing the multi-source images from multiple sensors into a synthesized image that provides either comprehensive or reliable description, and quality improvement technique can be used to address the challenge of low-quality image analysis task (Jin et al., 2017, 2021, 2023; Chen et al., 2021; Wang et al., 2022; Jiang et al., 2023). At present, a lot of brain-inspired algorithms methods (or models) are aggressively proposed to accomplish these two tasks, and the artificial neural network has become one of the most popular techniques in processing image fusion and quality improvement techniques in this decade, especially deep convolutional neural networks (Chen et al., 2021; Jin et al., 2021, 2023). This is an exciting research field for the research community of image fusion and there are many interesting issues remain to be explored, such as deep few-shot learning, unsupervised learning, application of embodied neural systems, and industrial applications.

How to develop a sound biological neural network and embedded system to extract the multiple features of source images are basically two key questions that need to be addressed in the fields of image fusion and quality improvement. Hence, studies in this field can be divided into two aspects: first, new end-to-end neural network models for merging constituent parts during the image fusion process; Second, the embodiment of artificial neural networks for image processing systems. In addition, current booming techniques, including deep neural systems and embodied artificial intelligence systems, are considered as potential future trends for reinforcing the performance of image fusion and quality improvement.

In the first work entitled “*Multi-focus image fusion dataset and algorithm test in real environment*,” Liu S et al. proposed a multi-focus image fusion dataset named HBU-CVMDSP. The dataset can truly reflect the real-world scene, which included 66 groups of images captured by smartphones. Five image fusion algorithms were performed on the HBU-CVMDSP dataset, which revealed that the HBU-CVMDSP dataset could better promote the research of multi-focus image fusion.

Due to insufficient view refinement feature extraction and poor generalization ability of the network model affecting the classification accuracy, Wang et al. proposed a multi-view SoftPool attention convolutional network for 3D model classification tasks. The multi-view features were extracted through ResNest and adaptive pooling modules, and then processed by SoftPool, which enabled the subsequent refinement extraction. The experimental results showed that the proposed model is effective.

In the third paper, Kong et al. proposed the model of convolutional extreme learning machine (CELM) for the fusion of multimodal medical images. In this method, CELM served as an important tool to extract and capture the features of source images from a variety of different angles, and the final fused image can be obtained by integrating the significant features. Experiments showed that the proposed method has obvious superiorities in gray image fusion and color image fusion.

The visual quality of images will be seriously affected by bad weather conditions, especially on foggy days. Yang et al. proposed a new transformer-based progressive residual network (PRnet) to achieve the quality improvement and obtain a fog-free image. In this work, the swin transformer block encoded the feature representation of the decomposed block and continuously reduced the feature mapping resolution. The decoder was used to recursively select and fuse image features. Experiments showed that the performance of the proposed method was better than other state-of-the-art methods.

Zhang C et al. proposed a lightweight multi-dimensional dynamic convolutional network (LMDCNet) for real-time semantic segmentation with an ideal trade-off between model parameters, segmentation accuracy and inference speed. In this work, the encoder was a depth-wise asymmetric bottleneck module with multi-dimensional dynamic convolution and shuffling operations (MS-DAB), which increased the utilization of local and contextual information of features. Finally, a feature pyramid module (SC-FP) based on spatial and channel attention can perform the multi-scale fusion of features accompanied by feature selection.

Ye et al. proposed a dual branch CNN network (BD-CNN) for the fusion and classification of multi-source remote sensing data. Comparing with ELM algorithm and SVM algorithm, the proposed BD-CNN model can effectively fuse and classify multi-source remote sensing data.

Electricity transmission line monitoring in hazy weather will face some problems, such as reduced contrast and chromatic aberration. Therefore, Zhang M et al. proposed an image defogging algorithm for the electricity transmission line monitoring system. In this research, an optimized quadtree segmentation method for calculating global atmospheric light was proposed. Moreover, the detail sharpening post-processing based on visibility and air light level was introduced to enhance the detail level of electricity transmission lines in the defogging image. Experiments proved that the algorithm performs well in improving image quality.

Chen et al. proposed an improved multi-exposure fusion method based on the exposure fusion framework and the color dissimilarity feature to solve the problem of ghosting artifacts. First, an improved exposure fusion framework based on the camera response model was applied to preprocess the input image sequence. Then, an improved color dissimilarity feature was used to detect the object motion features in dynamic scenes. Finally, the improved pyramid model was adopted to retain detailed information about the poor exposure areas.

To preserve more local details and with few artifacts in panoramas, Tang et al. presented an improved mesh-based joint optimization image stitching model. An improved energy function containing a color similarity term and a regularization parameter strategy of combining the proposed method with an as-projective-as-possible (APAP) warp was performed. Moreover, calculating the distance between the vertex and the nearest matched feature point to the vertex ensured a more natural stitching effect in non-overlapping areas.

The 1D convolution is not limited by the input size and has the advantage of fewer parameters. Thus, Zhang C et al. designed a lightweight semantic segmentation network (LSNet) composed of full 1D convolution. Moreover, increasing the depth of network in the decoder can effectively solve the misalignment of upsampling and improve the accuracy of network segmentation. Experiments demonstrated that the proposed method can achieved better performance in accuracy and parameters.

As most attack methods rely on a relatively loose noise budget in image, Liu R et al. proposed a novel framework named Dual-Flow for generating adversarial examples by disturbing the latent representation of the clean examples. The spatial transform techniques were applied to the latent value to preserve the details of original images and guarantee the adversarial images' quality. Experiments revealed the superiority of the proposed method in synthesizing adversarial examples.

Mi et al. proposed a deep learning algorithm based on the modified YOLOv4 network to improve the accuracy of railway defects detection. In this mehod, the rail region extraction, improved Retinex image enhancement, background modeling difference and threshold segmentation were performed sequentially to obtain the segmentation map of defects. For the classification of defects, Res2Net and Convolutional Block Attention Module (CBAM) were introduced to improve the receptive field and small target position weights.

Shi et al. proposed an evaluation system based on image quality indexes, resource occupancy and energy consumption metrics, which verified the performances of different near-infrared image colorization methods on low-power NVIDIA Jetson embedded systems. Eleven infrared image colorization methods were tested on three different configurations of NVIDIA Jetson boards. The experimental results indicated that the CICZ had the smallest energy consumption per unit of time. Pix2Pix and TIC-CGAN showed superiority in image quality and latency metrics. Moreover, the RecycleGAN, PearlGAN and I2V-GAN had smaller memory usage than other methods on edge devices.

## Author contributions

All authors listed have made a substantial, direct, and intellectual contribution to the work and approved it for publication.
